# Draft Genome Sequence of a Potential Nitrate-Dependent Fe(II)-Oxidizing Bacterium, *Aquabacterium parvum* B6

**DOI:** 10.1128/genomeA.01651-15

**Published:** 2016-01-28

**Authors:** Xiaoxin Zhang, Fang Ma, Ulrich Szewzyk

**Affiliations:** a State Key Laboratory of Urban Water Resource and Environment, School of Municipal and Environmental Engineering, Harbin Institute of Technology, Harbin, China; b Department of Environmental Microbiology, Technical University of Berlin, Berlin, Germany

## Abstract

*Aquabacterium parvum* B6 is a potential nitrate-dependent Fe(II)-oxidizing bacterium. The genes related to its denitrifying mechanism and iron metabolisms were unknown. We present the draft genome of *Aquabacterium parvum* B6, which could provide further insight into the nitrate-dependent Fe(II)-oxidizing mechanism of strain B6.

## GENOME ANNOUNCEMENT

Nitrate-dependent Fe(II)-oxidizing (NDFO) bacteria, conducting nitrate-dependent Fe(II)-oxidation, include both autotrophic and heterotrophic organisms (1, 2). In previous studies, several *Aquabacterium* spp. have been mentioned as capable of NDFO. A ferrous iron-oxidizing, nitrate-reducing bacterium, *Aquabacterium* strain BrG2, was obtained from freshwater sediments (3). The strain is facultative anaerobic, and the Fe(II) oxidation could be accelerated significantly with the presence of an organic co-substrate (4). A short rod-shaped *Aquabacterium fontiphilum* sp. nov., isolated from springwater in Taiwan, was also reported to accomplish nitrate reduction via utilizing nitrate serves as an alternative electron acceptor (5). Moreover, isolates from the sediments in the littoral of Lake Constance closely related to the *Aquabacterium* cluster were also able to conduct NDFO (6). To provide clues for future investigations of the mechanism and metabolism of *Aquabacteria*, as well as to facilitate further studies with the genus and species, we report here a draft genome assembly of *Aquabacterium parvum*.

*Aquabacterium parvum* strain B6, characterized approximately two decades ago, is a Gram-negative, rod-shaped bacterium, isolated from a Berlin drinking water distribution system (7). The strain is available from the German Collection of Microorganisms and Cell Cultures (Deutsche Sammlung von Mikroorganismen und Zellkulturen, DSMZ 11968). Draft genome data for strain B6 were determined using the Illumina HiSeq2000 system. The reads were assembled into 619 large contigs using Velvet version 1/2/10 (8). The contig *N*_50_ is approximately 10.7 kb, and the largest contig assembled is approximately 50.0 kb. The draft sequence consists of 4,592,999 bases with a mean G+C percentage of 65.3%. Annotation of open reading frames was performed by using the RAST annotation server (9) and the NCBI Prokaryotic Genome Annotation Pipeline (10). A total of 4,019 coding sequences and 63 structural RNAs were predicted. There are 429 subsystems in the genome, as determined by the RAST server. Comparison with the genome sequences available at the RAST server suggested that the closest neighbor of strain B6 is *Leptothrix cholodnii* SP6 (score 533), followed by *Methylibium petroleiphilum* PM1 (score 528), *Alicycliphilus denitrificans* BC (score 389), and *Acidovorax* sp. JS42 (score 383).

### Nucleotide sequence accession numbers.

This whole-genome shotgun project has been deposited at DDBJ/EMBL/GenBank under the accession number LFRI00000000. The version described in this paper is the first version, LFRI01000000.
